# Rapamycin promotes endothelial–mesenchymal transition during stress-induced premature senescence through the activation of autophagy

**DOI:** 10.1186/s12964-020-00533-w

**Published:** 2020-03-12

**Authors:** Norihiko Sasaki, Yoko Itakura, Masashi Toyoda

**Affiliations:** Department of Geriatric Medicine (Vascular Medicine), Tokyo Metropolitan Institute of Gerontology, Sakaecho 35-2, Itabashi-ku, Tokyo, 173-0015 Japan

**Keywords:** Rapamycin, Senescence-associated secretory phenotype, Endothelial cells, Stress-induced premature senescence, Endothelial-mesenchymal transition, Autophagy

## Abstract

**Background:**

Rapamycin is known to be effective in suppressing senescence and the senescence-associated secretory phenotype (SASP). Therefore, it is highly expected to represent an anti-aging drug. Its anti-aging effect has been demonstrated at the mouse individual level. However, there are not many clinical findings with respect to its activity in humans. Here, we aimed to clarify the effect of rapamycin on human endothelial cells (ECs) as an in vitro model of human blood vessels.

**Methods:**

Over the course of oxidative stress-induced senescence using hydrogen peroxide, we examined the effect of rapamycin on human coronary artery ECs (HCAECs). Senescence was evaluated by detecting senescence-associated β-galactosidase (SA-β-Gal) activity and the real-time PCR analysis of *p16*^*INK4a*^. Furthermore, expression levels of SASP factors were examined by real-time PCR and the expression of senescence-related antigens, such as intercellular adhesion molecule-1 (ICAM-1) and ganglioside GM1, were examined by fluorescence-activated cell sorting analysis and immunostaining. The inhibitory effect of rapamycin on mTOR signaling was examined by immunoblotting. The adhesion of leukocytes to HCAECs was evaluated by adhesion assays. Endothelial–mesenchymal transition (EndMT) induced by rapamycin treatment was evaluated by real-time PCR analysis and immunostaining for EndMT markers. Finally, we checked the activation of autophagy by immunoblotting and examined its contribution to EndMT by using a specific inhibitor. Furthermore, we examined how the activation of autophagy influences TGF-β signaling by immunoblotting for Smad2/3 and Smad7.

**Results:**

A decrease in SA-β-Gal activity and the suppression of SASP factors were observed in HCAECs undergoing stress-induced premature senescence (SIPS) after rapamycin treatment. In contrast, ICAM-1 and ganglioside GM1 were upregulated by rapamycin treatment. In addition, leukocyte adhesion to HCAECs was promoted by this treatment. In rapamycin-treated HCAECs, morphological changes and the promotion of EndMT were also observed. Furthermore, we found that autophagy activation induced by rapamycin treatment, which led to activation of the TGF-β pathway, contributed to EndMT induction.

**Conclusions:**

We revealed that although rapamycin functions to inhibit senescence and suppress SASP in HCAECs undergoing SIPS, EndMT is induced due to the activation of autophagy.

Video abstract

## Background

Vascular endothelial cells (ECs), which constitute the endothelium of blood vessels, play pivotal roles in vascular homeostasis. The excessive activation or dysfunction of ECs leads to the development of vascular-related diseases such as restenosis and arteriosclerosis [1]. The aging worldwide population is resulting in an increased number of elderly individuals, among whom vascular disease is the leading cause of death. EC senescence is considered to increase the risk of vascular diseases [2, 3]. Therefore, it is important to clarify the mechanisms underlying senescence-associated diseases to lower the risk for vascular disease and extend healthy life expectancy.

Senescent cells exhibit an arrest in proliferation but also changes in protein expression and secretion that comprise the senescence-associated secretory phenotypes (SASPs) [4]. In ECs, intercellular adhesion molecule-1 (ICAM-1), which is a major adhesion molecule that plays a critical role in the homing of leukocytes to sites of atherosclerotic lesions, is known to be induced by senescence [5]. ICAM-1 expression is implicated in the formation and progression of atherosclerotic lesions [6, 7], and therefore, senescence is thought to promote ICAM-1-mediated vascular diseases [2]. Previously, we reported that senescent ECs exhibit the increased expression of ganglioside GM1, a glycolipid that is localized to the cell membrane, and that increased GM1 contributes to vascular insulin resistance, which is considered to play an important role in the pathogenesis of vascular-related diseases including atherosclerosis [8–10]. The SASP reinforces senescence and activates an immune surveillance response, but it can also influence the tumor microenvironment and contribute to age-related pathologies such as vascular disease [11–14]. To date, research targeting senescent cells has been conducted extensively, and it was demonstrated that senotherapies target senescent cells for death either by apoptosis (i.e. senotopsis) or non-apoptotic death (i.e. senolysis) and that the removal of senescent cells in mice improves age-related diseases and extends the healthy life span, indicating the importance of SASP and senescence-related antigens in senescence-associated diseases [15–18]. Thus, it is considered that inhibiting the SASP in senescent ECs, which is effective for all vascular-related diseases in the body, is particularly important.

It has been shown that the immunosuppressive and anticancer drug rapamycin can prevent cellular and organismal aging. Rapamycin inhibits the activity of the two mammalian target of rapamycin (mTOR) complexes, namely mTOR complex 1 (mTORC1), in which mTOR and RAPTOR are included [19], and mTOR complex 2 (mTORC2), in which mTOR and RICTOR are included [20]. mTORC1 stimulates transcription inducing SASP factor expression, ribosome biogenesis, translation initiation, nutrient uptake, and autophagy inhibition [21, 22]. In contrast, the role of mTORC2 is well known with respect to various cellular processes such as cytoskeleton rearrangement, glucose metabolism, and cell migration [23–25]. Inhibition of the TOR pathway by rapamycin extends the lifespan in several species such as yeast, fruit flies, and mice [26–29]. Using a variety of cell lines, several studies have shown that rapamycin also delays cellular senescence, as assessed by cell numbers, senescence-associated β-galactosidase (SA-β-Gal) staining, p16^INK4a^ expression, and inhibition of the SASP [30, 31]. Moreover, in ECs, the effects of rapamycin have been reported. For example, one study showed that rapamycin inhibits the tumor necrosis factor-induced expression of vascular cell adhesion molecule-1 in human umbilical vein ECs (HUVECs) [32], whereas another study showed that pretreatment with rapamycin dose-dependently inhibits ox-LDL-induced increases in adhesion molecule expression and macrophage adhesion to HUVECs [33]. Further, it has been shown that rapamycin decreases rat EC proliferation [34].

However, to date, the effects of rapamycin on ECs during the induction of senescence have not been extensively elucidated. Accordingly, we investigated the effect of rapamycin on stress-induced premature senescence (SIPS) in ECs. For this, we examined senescence, the SASP, expression of senescence-related cell surface antigens (ICAM-1 and GM1), and EC function after treatment with rapamycin.

## Materials and methods

### Cell culture

Human coronary artery ECs (HCAECs) were purchased from a commercial vendor (Lonza, Walkersville, MD, USA). For the initial culture (baseline), these cells did not express the smooth muscle marker, smooth muscle myosin heavy chain. HCAECs were grown in EGM-2MV (Lonza) with added supplements and growth factors. U937 cells, a human monocytic cell line, were maintained in RPMI 1640 medium with 10% FBS, 1% penicillin/streptomycin, and 2 mM L-glutamine. HCAECs were passaged at 80% confluence and seeded at a density of 2500–5000 cells/cm^2^. HCAECs were studied at a confluence of 75–80%. Population doubling levels (PDLs) were calculated at each passage using the following equation: n = (log_2_X − log_2_Y) (where n = the PDL, X = the number of cells at the end of one passage, and Y = the number of cells that were seeded at the beginning of one passage). The PDL at the first plating of a newly purchased cell stock was defined as PDL 0. In this study, HCAECs with PDL 11–15 were used as early-passage (non-senescent) cells. For the induction of premature senescence, early-passage cells at approximately 75–80% confluence were exposed to 250 μM hydrogen peroxide (H_2_O_2_) (Sigma-Aldrich Corporation, St. Louis, MO, USA) diluted in culture medium for 2 h. The cells were washed three times with PBS to remove H_2_O_2_ and re-cultured in fresh culture medium for 72 h to facilitate the appearance of senescent characteristics. Rapamycin (LC Laboratories Inc., Woburn, MA, USA) treatment was performed as shown in figure legends. To inhibit autophagy, HCAECs were pretreated with 100 nM bafilomycin A1 (Sigma-Aldrich) for 1 h before rapamycin treatment. 0.0001% DMSO was used as vehicle control.

### SA-β-gal assay

SA-β-Gal activity was assayed using a senescence detection kit (BioVision Inc., Milpitas, CA, USA) as described in our previous reports [8]. Briefly, cells were washed twice with PBS, exposed to fixation solution for 10 min, and then incubated overnight in freshly prepared staining solution. After staining, cells were counterstained with DAPI and then four fields of view were examine microscopically. By counting the number of SA-β-Gal-positive cells based on their blue color and the total number of cells stained with DAPI using ImageJ software (National Institutes of Health, Bethesda, MD, USA), the percentage of SA-β-Gal-positive cells was calculated to estimate the percentage of senescent cells.

### Fluorescence-activated cell sorting (FACS) analysis

As described in our previous reports [8, 9], cells were harvested with the Accutase® cell detachment solution (Merck Millipore, Billerica, MA, USA) and dissociated single cells were incubated with FITC-conjugated ICAM-1 (BioLegend, San Diego, CA, USA) or FITC-isotype control (Becton Dickinson, Franklin Lakes, NJ, USA) diluted in FACS buffer (0.5% [w/v] BSA and 0.1% [w/v] sodium azide in PBS) for 30 min on ice. To detect GM1, cells were incubated with Alexa Fluor® 647-conjugated cholera toxin B subunit (Molecular Probes, Eugene, OR, USA) diluted in FACS buffer for 30 min on ice. After washing, cell sorting and analysis were performed using a FACSAria™ Cell Sorter (Becton Dickinson). Mean fluorescence intensities (MFIs) were calculated by subtracting the intensities of the controls.

### Cell cycle assay

Cells were washed with PBS, resuspended in PBS, and stained with the cell cycle assay solution (Deep Red; Dojindo Molecular Technologies, Inc., Rockville, MD, USA) followed by incubation at 37 °C for 15 min, according to the manufacturer’s protocol. The cell cycle profiles were obtained using a FACSAria cell sorter at 640 nm, and the data were analyzed using FlowJo software (Becton Dickinson).

### Immunoblotting

Cells were lysed with lysis buffer (50 mM Tris-HCl pH 7.4, 150 mM NaCl, 1.5 mM MgCl_2_, 5 mM EDTA, and 1% Triton™ X-100) containing protease and phosphatase inhibitor cocktails and immunoprecipitations were performed with monoclonal rabbit anti-mTOR (ab32028; Abcam, Cambridge, UK) and protein G magnetic beads (Veritas, Tokyo, Japan). Samples prepared as described previously herein were separated by SDS-PAGE using a gel of the appropriate percentage and then transferred onto PVDF membranes (Merck Millipore). After blocking, the membranes were incubated with the following primary antibodies: monoclonal rabbit anti-mTOR (dilution 1:1000), polyclonal rabbit anti-RICTOR (dilution 1:1000; Proteintech Group, Inc., Rosemont, IL, USA), monoclonal rabbit anti-RAPTOR (dilution 1:1000; ab40768; Abcam), monoclonal rabbit anti-p-Smad3 (dilution 1:1000; ab52903; Abcam), monoclonal rabbit anti-Smad2/3 (dilution 1:1000; #8685; Cell Signaling Technology, Danvers, MA, USA), polyclonal rabbit anti-TGFβR-I (dilution 1:1000; SAB4502958; Sigma-Aldrich, St. Louis, MO, USA), monoclonal rabbit anti-TGFβR-II (dilution 1:1000; ab184948; Abcam), polyclonal rabbit anti-Smad7 (dilution 1:1000; Proteintech), polyclonal rabbit anti-MAPKAPK2 (dilution 1:1000; #3042; Cell Signaling Technology), monoclonal rabbit anti-LC3 (dilution 1:1000; ab192890; Abcam) and monoclonal mouse anti-β-ACTIN (dilution 1:10000; A5316; Sigma-Aldrich). The membranes were then incubated with the appropriate peroxidase-conjugated secondary antibodies (dilution 1:30000; Cell Signaling Technology), washed, and developed with ECL™ Prime reagents (GE Healthcare, Piscataway, NJ, USA). Immunoblot images were densitometrically analyzed using ImageJ software.

### ELISA assay

The cell culture supernatants were analyzed for active human TGF-β1 levels by ELISA kit (Cloud-Clone Corp., Katy, TX, USA), following the manufacturer’s protocol. The productivity rate was calculated from the concentration of TGF-β1 with respect to the cell number.

### Real-time PCR

Total RNA was isolated from cells using the RNeasy plus mini kit (QIAGEN, Hilden, Germany) and subsequently reverse-transcribed using the ReverTra Ace® qPCR RT Kit (Toyobo, Osaka, Japan). Real-time PCR was performed using the Power Sybr® Green kit (Applied Biosystems, Foster City, CA, USA) and the StepOnePlus™ real-time PCR system (Applied Biosystems). *β-ACTIN* was amplified and used as an internal control. The threshold crossing value was noted for each transcript and normalized to that of the internal control. Relative quantitation of each mRNA was performed using the comparative Ct method. Primer sets for real-time PCR are listed in Table 1.

**Table 1:** List of primer sets for real-time PCR

Gene	Forward primer	Reverse primer
*αSMA*	CACCATCGGAAATGAACGTTT	GACTCCATCCCGATGAAGGA
*SM22*	GGCGTGATTCTGAGCAAGCT	CACCTTCACCGGCTTGGA
*FSP1*	TGGAGAAGGCCCTGGATGT	CCCTCTTTGCCCGAGTACTTG
*vWF*	CGGCTTGCACCATTCAGCTA	TGCAGAAGTGAGTATCACAGCCATC
*p16*	CCAACGCACCGAATAGTTACG	GGGCGCTGCCCATCA
*IL-1α*	TGGAGGCCATCGCCAAT	AGGAAGCTAAAAGGTGCTGACCTA
*IL-1β*	GTCTGGTCCATATGAACTGAAAGCT	GGACATGGAGAACACCACTTGTT
*IL-8*	ACTGAGAGTGATTGAGAGTGGAC	AACCCTCTGCACCCAGTTTTC
*GROα*	CCACTGCGCCCAAACC	GCAAGCTTTCCGCCCATT
*TNFα*	CCCAGGCAGTCAGATCATCTTC	GCTTGAGGGTTTGCTACAACATG
*MCP1*	GAAGAATCACCAGCAGCAAGTG	GATCTCCTTGGCCACAATGG
*GMCSF*	GAGCATGTGAATGCCATCCA	TTCATTCATCTCAGCAGCAGTGT
*β-ACTIN*	GGTCATCACCATTGGCAATGAG	TACAGGTCTTTGCGGATGTCC

### Monocyte adhesion assay

One day before the assay, HCAECs were plated at a density of 5 × 10^4^ cells in a 96-well plate. U937 cells were stained with LeukoTrackerTM (Cell Biolabs, Inc., San Diego, CA, USA) for 60 min at 37 °C. LeukoTrackerTM-labeled U937 cells (5 × 10^5^ cells/ml) were suspended in serum-free RPMI 1640 medium and added to the HCAEC monolayer. After 60 min incubation at 37 °C, unattached cells were removed by careful washing with wash buffer (Cell Biolabs) and then the cells were lysed with lysis buffer (Cell Biolabs). Fluorescence was measured with a fluorescence plate reader (PerkinElmer, Inc., Waltham, MA, USA) at 480/520 nm.

### Immunostaining

Cells were fixed with 4% (w/v) paraformaldehyde and washed. Subsequently, cells were permeabilized with 0.1% [v/v] Triton™ X-100 and blocked with PBS containing 1% (w/v) BSA and 5% (v/v) normal goat serum. After washing, cells were incubated with anti-ICAM-1 (BioLegend) and anti-SM22 (ab14106, Abcam) antibodies at 4 °C overnight. After washing, cells were stained with Alexa Fluor® 488- and Alexa Fluor® 546-conjugated secondary antibodies (Molecular Probes) and Alexa Fluor® 647-conjugated cholera toxin B subunit for GM1 detection, and then counterstained with DAPI. Immunofluorescence images were acquired using a confocal laser scanning microscope. For the quantification of SM22-positive cells, the numbers of SM22-positive cells with red color and the total numbers of cells stained with DAPI from four fields were counted and then the percentage of SM22-positive cells was calculated.

### Statistical analysis

Results are expressed as means ± SD from at least three independent experiments. Statistical analysis was performed using EZR (Saitama Medical Centre, Jichi Medical University; http://www.jichi.ac.jp/saitama-sct/SaitamaHP.files/statmedEN.html; Kanda, 2012). One-way ANOVAs were performed to compare multiple groups.

## Results

### SA-β-gal activity is inhibited by rapamycin in ECs undergoing SIPS

**Fig. 1:**
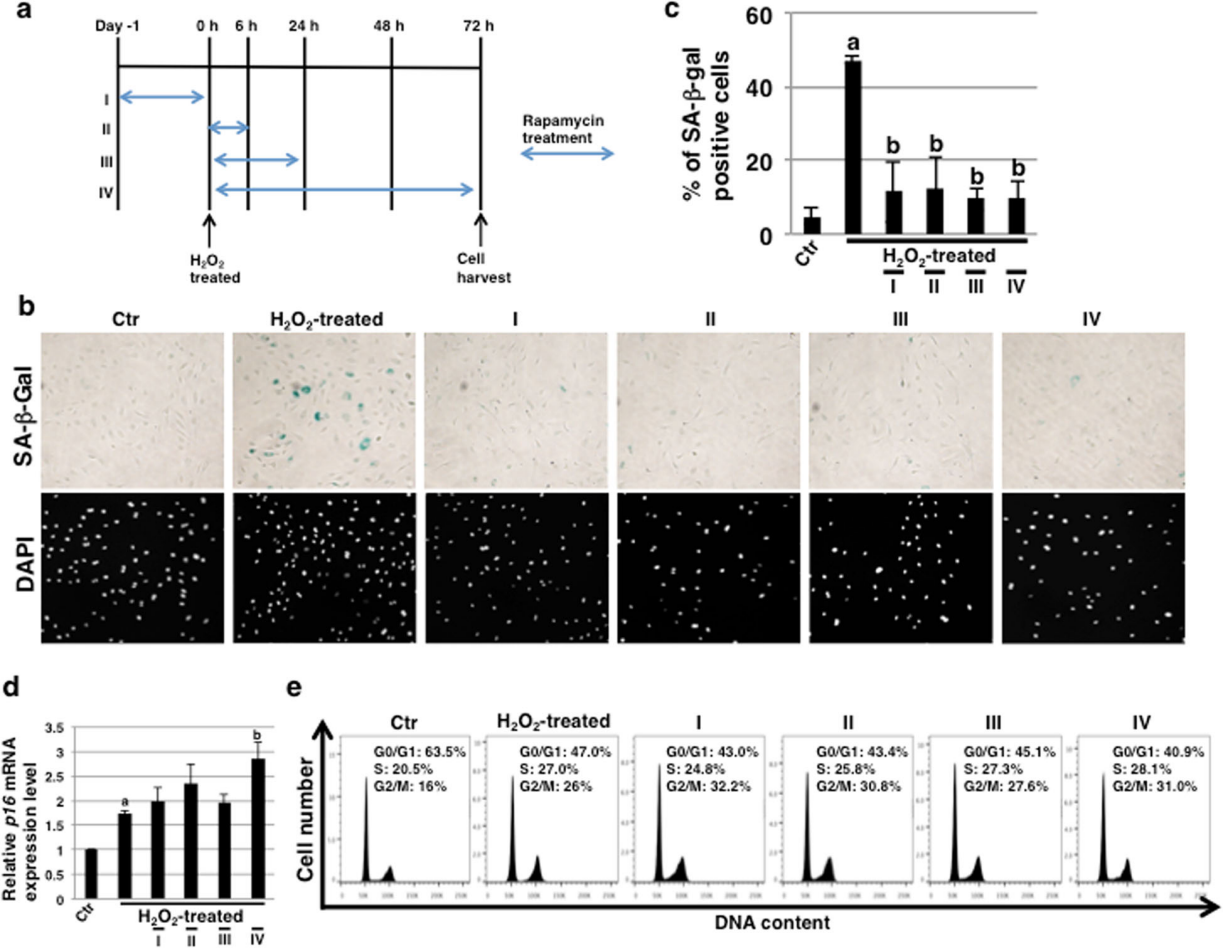
SA-β-Gal activity is inhibited by rapamycin in endothelial cells (ECs) subjected to SIPS. **a** Schedule of SIPS-induction, rapamycin treatment and EC culture. I: rapamycin pretreatment before H_2_O_2_ treatment, II: 6 h rapamycin treatment, III: 24 h rapamycin treatment, IV: 72 h rapamycin treatment. In II-IV experiments, rapamycin was added 2 h after H_2_O_2_ treatment. **b** Human coronary artery endothelial cells (HCAECs) at 72 h in (**a**) were stained for SA-β-Gal activity. Representative images of staining for SA-β-Gal and DAPI are shown. **c** SA-β-Gal-positive cells in (**b**) were quantitated as a percentage of total cell numbers. **d** Real-time PCR analysis of *p16*^*INK4a*^ using cDNA derived from HCAECs at 72 h in (**a**). The results are shown after normalization against values obtained for control HCAECs (value = 1). Results are presented as means ± SD from three independent experiments. **e** Cell cycle analysis of HCAECs at 72 h in (**a**). Representative data are shown. ^a^*p* < 0.05 vs. the Ctr and ^b^*p* < 0.05 vs. the H_2_O_2_-treated HCAECs. Ctr Control (Ctr): untreated cells. SIPS, stress-induced premature senescence

The inhibition of mTORC1 by rapamycin occurs within minutes, whereas the inhibition of mTORC2 occurs after prolonged (> 24 h) treatment [35]. Thus, the inhibition of mTOR signaling by rapamycin is time-dependent, but the inhibitory mechanisms during SIPS in HCAECs have not been clarified yet. Then, we designed a schedule of rapamycin treatment as shown in Fig. 1a. The serum concentrations of rapamycin for clinical application are usually within 7 nM to 16 nM [36, 37]. In this study, we investigated whether 10 nM rapamycin, which is analogous to serum concentrations, can inhibit SIPS in HCAECs. Three days after SIPS, we examined the expression of SA-β-Gal activity, which is a well-known characteristic of senescent cells, to evaluate this process. We found that SA-β-Gal activity in rapamycin-treated HCAECs was clearly reduced with every schedule of rapamycin treatment (Fig. 1b and c), indicating that the effect of even short-term treatment on HCAECs is maintained. In contrast, expression levels of another senescence marker, *p16*^*INK4a*^, were not reduced but seemed to increase with every schedule of rapamycin treatment (Fig. 1d), suggesting that cell-cycle arrest in SIPS of HCAECs could not be rescued with rapamycin. Further, to validate the results, we performed cell cycle assay through FACS analysis. It was observed that the H_2_O_2_-treated HCAECs had a higher G2/M fraction than the control HCAECs (24.0 ± 1.8% vs. 12.2 ± 4.8%), wherein the rapamycin-treated HCAECs revealed a comparatively higher G2/M fraction than that of H_2_O_2_-treated HCAECs (I, 29.4 ± 1.8%; II, 28.1 ± 5.3%; III, 27.5 ± 3.7%; IV, 29.2 ± 4.7% vs. H_2_O_2_-treated, 24.0 ± 1.8%) (Fig. 1e). These results indicated that the cell-cycle arrest could not be rescued with rapamycin. Thus, these results demonstrate that rapamycin can inhibit SA-β-Gal activity but not the expression of *p16*^*INK4a*^ including cell-cycle arrest in HCAECs undergoing SIPS.

### SASP is inhibited by rapamycin in ECs undergoing SIPS

**Fig. 2:**
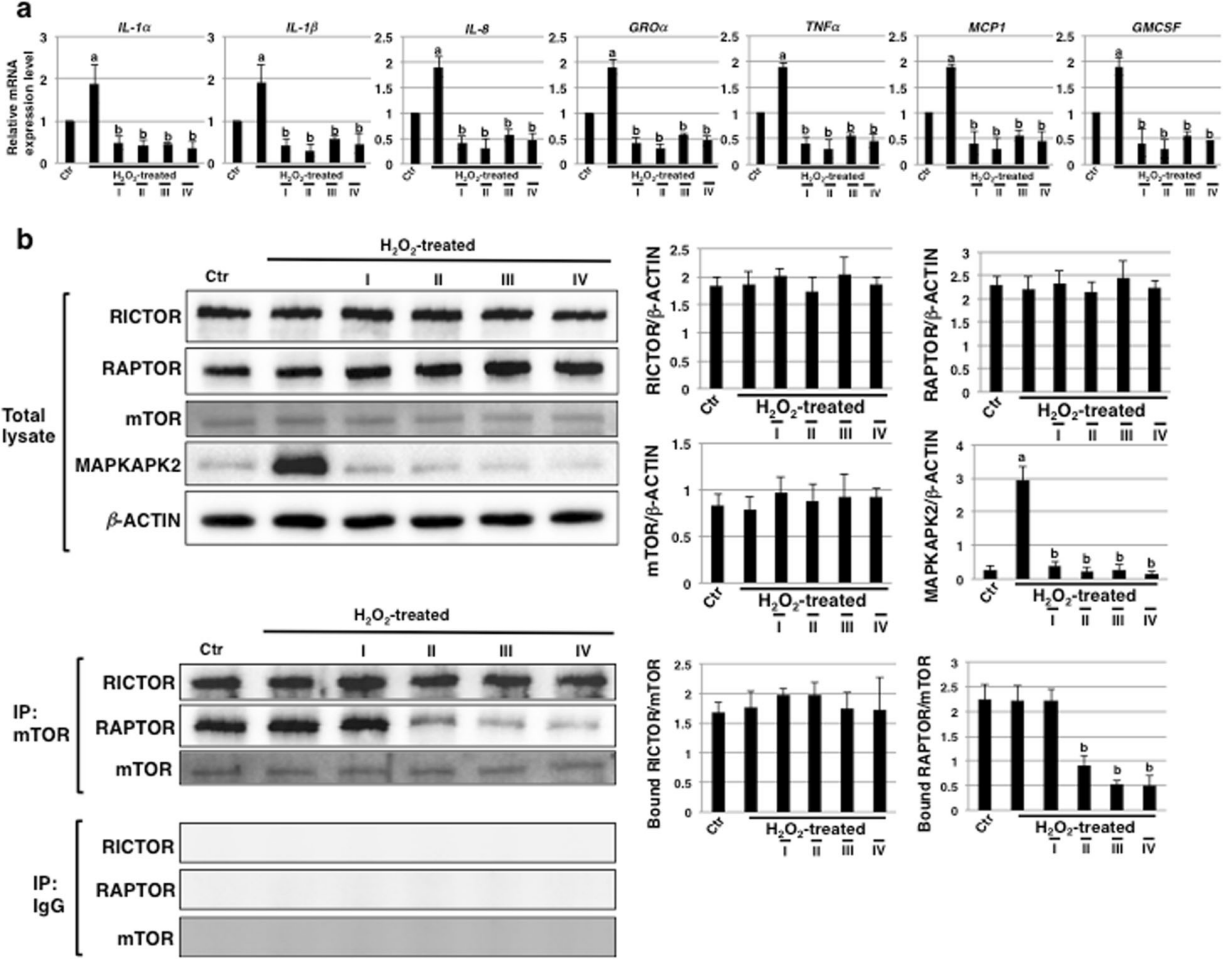
The SASP is inhibited by rapamycin in endothelial cells (ECs) subjected to SIPS. **a** Real-time PCR analysis of SASP factors using cDNA derived from human coronary artery endothelial cells (HCAECs) at 72 h in Fig. 1a. The results are shown after normalization to values obtained from control HCAECs (value = 1). Results are presented as means ± SD from three independent experiments. ^a^*p* < 0.05 vs. the Ctr and ^b^*p* < 0.05 vs. the H_2_O_2_-treated HCAECs. **b** Immunoblotting for the levels of mTOR, RICTOR, RAPTOR, and MAPKAPK2 was performed using cell lysates and mTOR immunoprecipitates from HCAECs at 72 h in Fig. 1a. β-ACTIN was used as a loading control. The histogram shows mean densitometric readings for the proteins normalized to β-ACTIN or mTOR from three independent experiments. ^a^*p* < 0.01 vs. the Ctr and ^b^*p* < 0.01 vs. the H_2_O_2_-treated HCAECs. Control (Ctr): untreated cells. SIPS, stress-induced premature senescence. SASP, senescence-associated secretory phenotype

Next, we performed real-time PCR analysis to determine the levels of SASP factors in HCAECs undergoing SIPS after rapamycin treatment. The levels of these including *IL-1α*, *IL-1β*, *IL-8*, *GROα*, *TNFα*, *MCP1*, and *GMCSF* were significantly upregulated with SIPS (Fig. 2a). However, in rapamycin-treated HCAECs, all examined SASP factors were drastically reduced with every schedule of rapamycin treatment (Fig. 2a). These results demonstrate that rapamycin treatment can inhibit SASP in HCAECs undergoing SIPS.

Then, we checked whether inhibition of the mTOR signaling pathway was apparent with every schedule of rapamycin treatment by investigating the levels of RAPTOR and RICTOR bound to mTOR by immunoblotting. The total expression levels of mTOR, RAPTOR, and RICTOR were not affected by rapamycin treatment (Fig. 2b, upper panel). Further, every schedule of rapamycin treatment did not attenuate the amounts of RICTOR bound to mTOR, indicating that the assembly of mTORC2 was not inhibited (Fig. 2b, lower panel). In contrast, the amounts of RAPTOR bound to mTOR were reduced in rapamycin-treated HCAECs in a time-dependent manner (Fig. 2b, lower panel), indicating that the reduced assembly of mTORC1 via mTOR inhibition continues for at least 6–72 h with rapamycin treatment after SIPS. In rapamycin-pretreated HCAECs, the amounts of RAPTOR bound to mTOR were reduced compared to the increased assembly of mTORC1 in H_2_O_2_-treated HCAECs at 24 h, as shown in Additional file 1: Figure S1a, right panel. This result indicates that the increase in mTOR signaling after H_2_O_2_ stress was attenuated in rapamycin-pretreated HCAECs at least within 24 h after H_2_O_2_ stress and that the inhibition of mTOR was restored at 72 h, as in Fig. S1a and Fig. 2b. The expression of SASP positive regulators such as *IL-1α* [38] and MAPKAPK2 [39], which are regulated by mTOR, were attenuated in rapamycin-pretreated HCAECs compared to the increased expression observed in H_2_O_2_-treated HCAECs at 24 h, as shown in Additional file 1: Figure S1a, left panel and b. Furthermore, the attenuation of MAPKAPK2 expression continued in rapamycin-pretreated HCAECs as well as with other schedule of rapamycin treatment (Fig. 2b, upper panel). Therefore, it was suggested that SASP inhibition in rapamycin-treated HCAECs undergoing SIP is dependent on the attenuation of *IL-1α* and MAPKAPK2 expression mediated by reduced assembly of mTORC1 via mTOR inhibition.

### Rapamycin treatment modulates senescence-related cell surface antigens in ECs undergoing SIPS

**Fig. 3:**
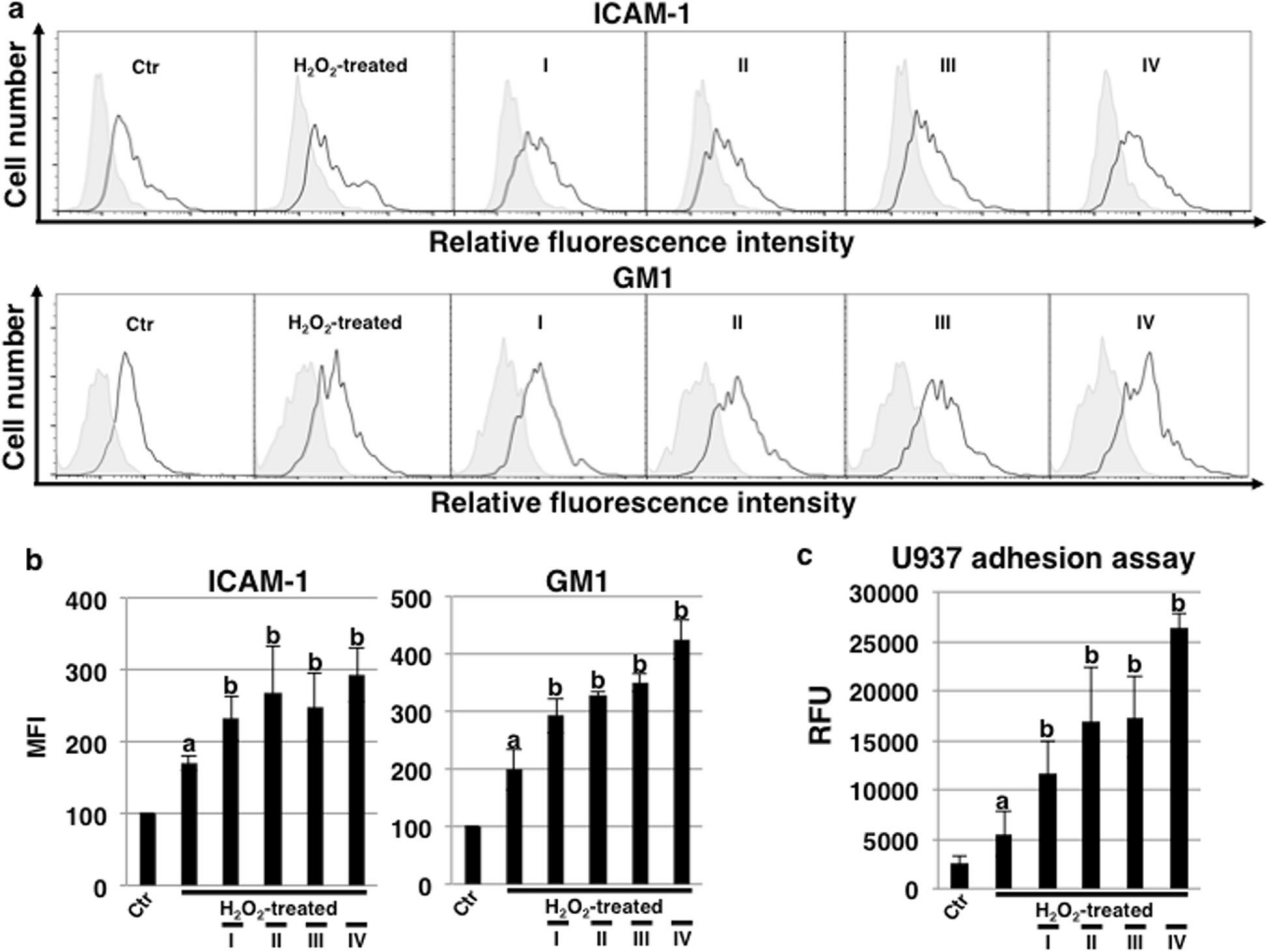
Rapamycin modulates senescence-related cell surface antigens and monocyte-adhesion. **a** FACS analysis of cell surface ICAM-1 and GM1 in human coronary artery endothelial cells (HCAECs) at 72 h in Fig. 1a. Representative results are shown. ICAM-1 and GM1 expression in HCAECs and negative controls are depicted by black dot lines and thin gray lines, respectively. **b** Mean fluorescence intensities (MFIs) relative to those in control HCAECs based on three independent experiments (**a**) are shown. **c** Adhesion assays with U937 cells were performed in HCAECs at 72 h, as in (**a**). ^a^*p* < 0.05 vs. the Ctr and ^b^*p* < 0.05 vs. the H_2_O_2_-treated HCAECs. Control (Ctr): untreated cells

We next investigated the expression of senescence-related cell surface antigens such as ICAM-1 and GM1 by FACS analysis. As a result, both ICAM-1 and GM1 increased with SIPS, and in cells cultured with rapamycin, a further increase in both antigens was observed for all culture conditions (Fig. 3a and b). The increase in cell adhesion via ICAM-1 was assumed from the increase in ICAM-1 and the increase in GM1 that might be related to the function of ICAM-1. Then, we examined the adhesion of inflammatory cells. Adhesion of the monocytic cell line U937 was increased in HCAECs undergoing SIPS, and adhesion was further increased with rapamycin treatment, concomitant with the increase in ICAM-1 and GM1 (Fig. 3c). Thus, we found that rapamycin affects the expression of ICAM-1 and GM1, leading to an increase in inflammatory cell adhesion.

### Rapamycin treatment alters the morphological features of ECs undergoing SIPS

**Fig. 4:**
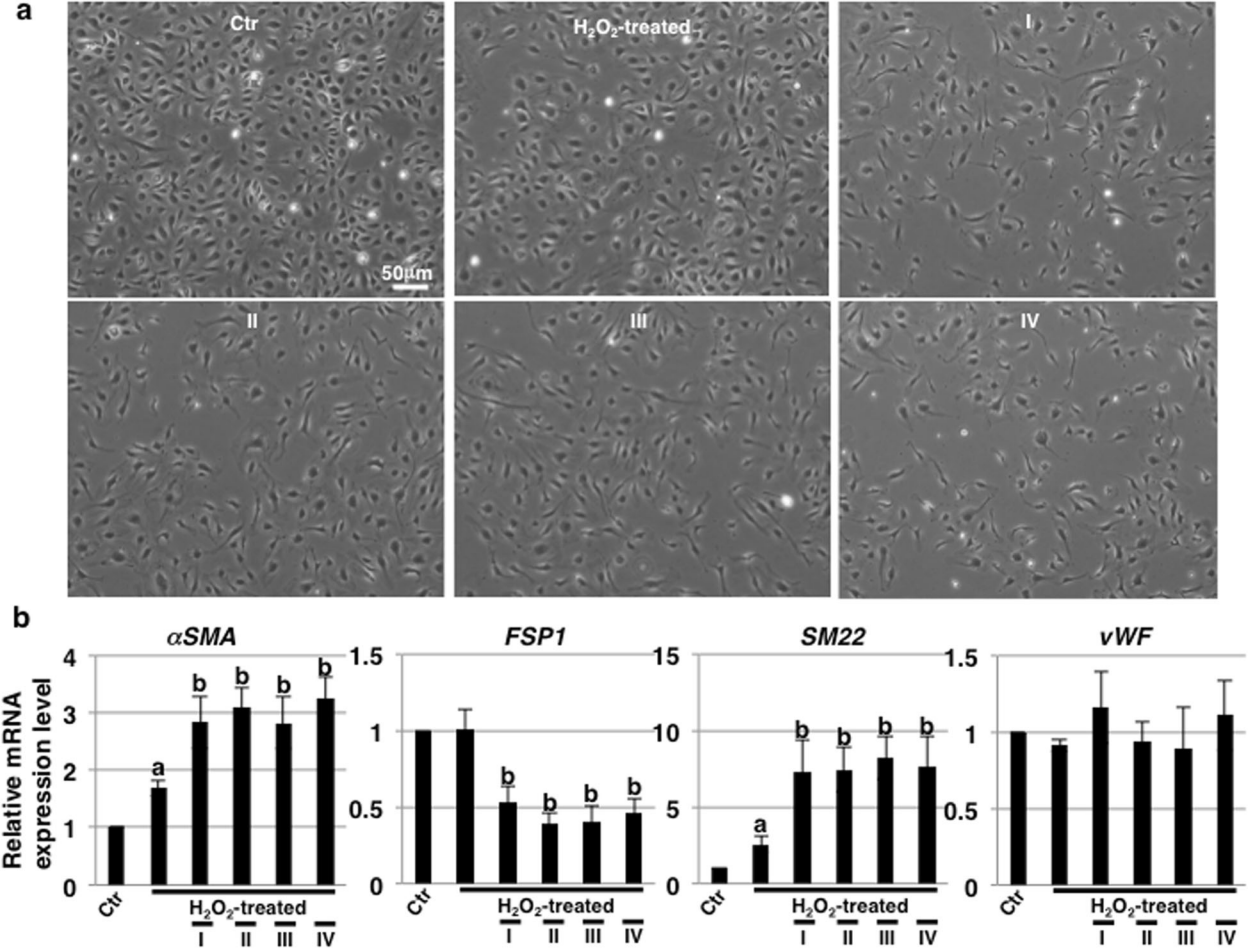
Rapamycin modulates endothelial cell morphological features. **a** Representative photos of human coronary artery endothelial cells (HCAECs) at 72 h in Fig. 1a. **b** Real-time PCR analysis of endothelial–mesenchymal transition (EndMT) markers using cDNA derived from HCAECs at 72 h in Fig. 1a. The results are shown after normalization to values obtained from control HCAECs (value = 1). Results are presented as means ± SD from three independent experiments. ^a^*p* < 0.05 vs. the Ctr and ^b^*p* < 0.05 vs. the H_2_O_2_-treated HCAECs. Control (Ctr): untreated cells

With rapamycin pretreatment overnight (0 h in Fig. 1a), there were no differences in morphology between control and rapamycin-treated HCAECs (data not shown). However, upon further culture (72 h), drastic changes in the morphology of rapamycin-treated HCAECs undergoing SIPS into elongated spindle-shaped form, which is a characteristic feature of the mesenchymal cells, were observed with every schedule of rapamycin treatment as compared to that in untreated cells subjected to SIPS (Fig. 4a). These results indicate that rapamycin has long-term morphology-altering effects on HCAECs undergoing SIPS. In ECs, endothelial–mesenchymal transition (EndMT) is induced by several exogenous agents such as TGF-β1, high glucose, and stress [40, 41]. It is known that H_2_O_2_ treatment contributes to the induction of mesenchymal and smooth muscle cell (SMC) differentiation of ECs, as were as EndMT [42]. Thus, we examined EndMT markers by real-time PCR analysis and found that these and SMC markers (*αSMA* and *SM22*) were upregulated in HCAECs subjected to SIPS (Fig. 4b). Further upregulation of these markers was observed in rapamycin-treated HCAECs with every schedule of treatment (Fig. 4b). In contrast, fibroblast marker (*FSP1*) was downregulated and not changed in one of EC marker (*vWF*) by rapamycin treatment (Fig. 4b). Thus, these results indicate that rapamycin also affects the morphology and EndMT induction, particularly partial differentiation into SM22-positive SMCs, in HCAECs undergoing SIPS.

### EndMT induction by rapamycin treatment is a consequence of autophagy activation

**Fig. 5:**
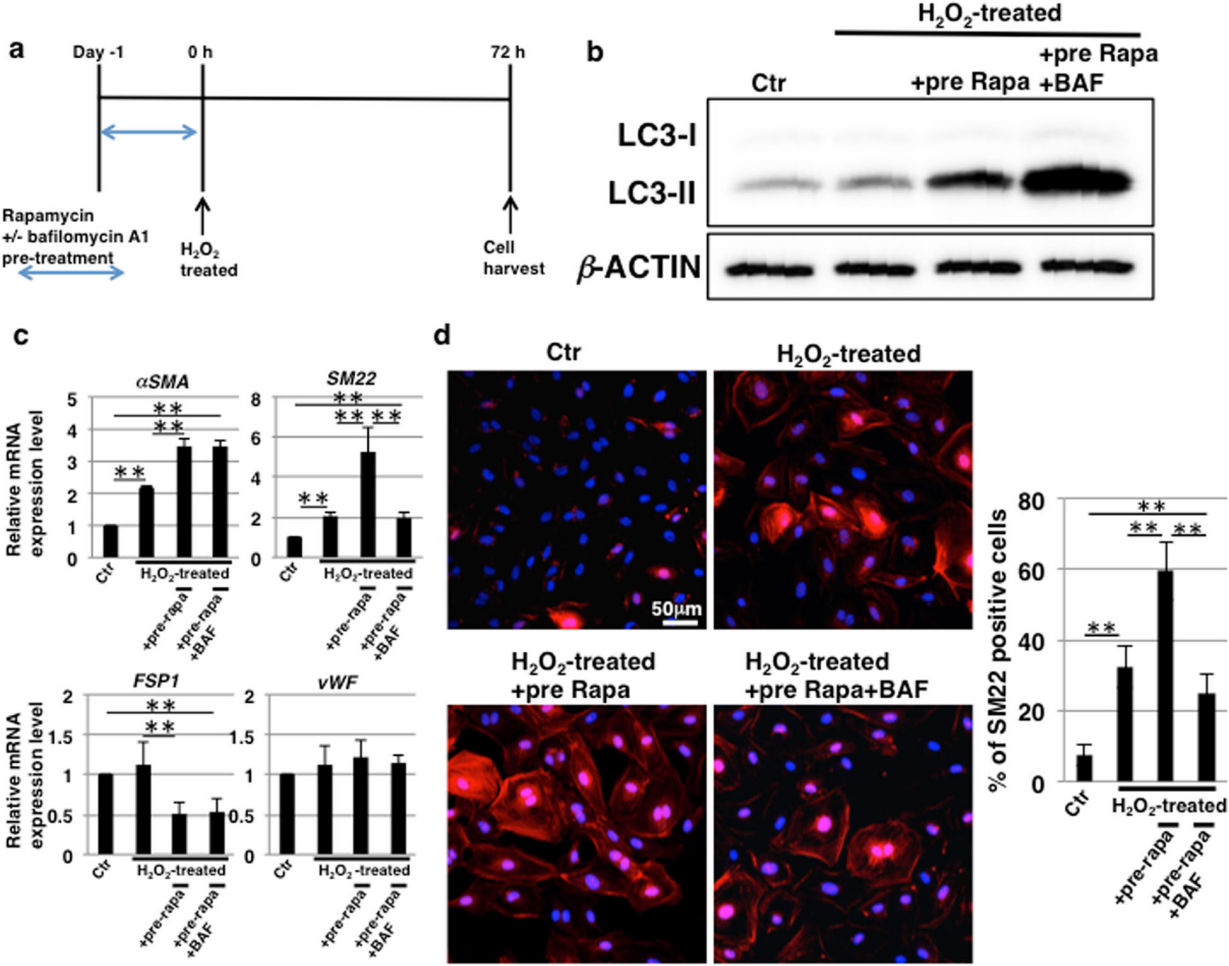
Induction of endothelial–mesenchymal transition (EndMT) by rapamycin treatment is caused by autophagy. **a** Schedule of stress-induced premature senescence (SIPS) induction, rapamycin treatment, and endothelial cell (EC) culture. To inhibit autophagy, human coronary artery endothelial cells (HCAECs) were pretreated with 100 nM bafilomycin A1 (BAF) for 1 h before rapamycin treatment. **b** Immunoblotting for the levels of LC3 was performed with cell lysates at day 0 in (**a**). β-ACTIN was used as a loading control. **c** Real-time PCR analysis of EndMT markers using cDNA derived from HCAECs at 72 h from (**a**). The results are shown after normalization to values obtained from control HCAECs (value = 1). Results are presented as means ± SD from three independent experiments. **p < 0.01. **d** Immunocytochemical staining performed in HCAECs at 72 h, as in (**a**). Representative images are shown (SM22, red; DAPI, blue). The histogram shows the mean ± SD percentage of SM22-positive cells. **p < 0.01. Control (Ctr): untreated cells

The inhibition of mTORC1 by rapamycin is known to induce autophagy, which is involved in cell differentiation and epithelial-mesenchymal transition [43–45]. Next, we investigated the possible involvement of autophagy in rapamycin-induced EndMT in HCAECs subjected to SIPS. From the previous results as above, every schedule of rapamycin treatment induced EndMT to the same extent and then we designed schedule of experiments as shown in Fig. 5a. First, we determined whether autophagy was induced by immunoblotting for LC3, which is a hallmark of autophagy. The conversion of LC3-I to LC3-II is associated with autophagy induction [46] and the amount of LC3-II is closely correlated with the number of autophagosomes [47]. As shown in Fig. 5b, the expression of LC3-II was upregulated in rapamycin-treated HCAECs compared to that in control and H_2_O_2_-treated HCAECs. Furthermore, treatment with bafilomycin A1, a widely-used inhibitor of autophagy, enhanced the expression of LC3-II (Fig. 5b). The use of bafilomycin A1 is known to increase LC3-II levels because of the accumulation of undigested autophagosomes [48]. Thus, it was confirmed that autophagy was induced after rapamycin treatment in HCAECs and that the activation was inhibited by treatment with bafilomycin A1. Bafilomycin A1 pretreatment had no effect on the repression of SA-β-Gal activity but attenuated the increased expression of *p16*^*INK4a*^ in rapamycin-treated HCAECs undergoing SIPS (Additional file 1: Figure S2a and b). Furthermore, bafilomycin A1 treatment had no effect on SASP repression (Additional file 1: Figure S2c). These results indicate that the activation of autophagy is unrelated to the repression of senescence and the SASP. In contrast, based on real-time PCR analysis for EndMT markers, we found that the increase in *SM22* expression induced by rapamycin treatment was inhibited by pretreatment with bafilomycin A1 (Fig. 5c). Furthermore, autophagy inhibition also suppressed the increase in ICAM-1 expression mediated by rapamycin treatment, whereas the increase in GM1 was enhanced (Additional file 1: Figure S3a). Immunostaining revealed that SM22-positive cells exhibited the co-expression of ICAM-1 and GM1 (Additional file 1: Figure S3b) and that the increase in SM22-positive cells induced by rapamycin treatment was inhibited by pretreatment with bafilomycin A1 in HCAECs subjected to SIPS (Fig. 5d). Taken together, it was suggested that the activation of autophagy by rapamycin treatment is involved in the induction of EndMT that accompanies ICAM-1 and GM1 expression in HCAECs undergoing SIPS.

### Rapamycin induces TGF-β pathway activation through autophagy

**Fig. 6:**
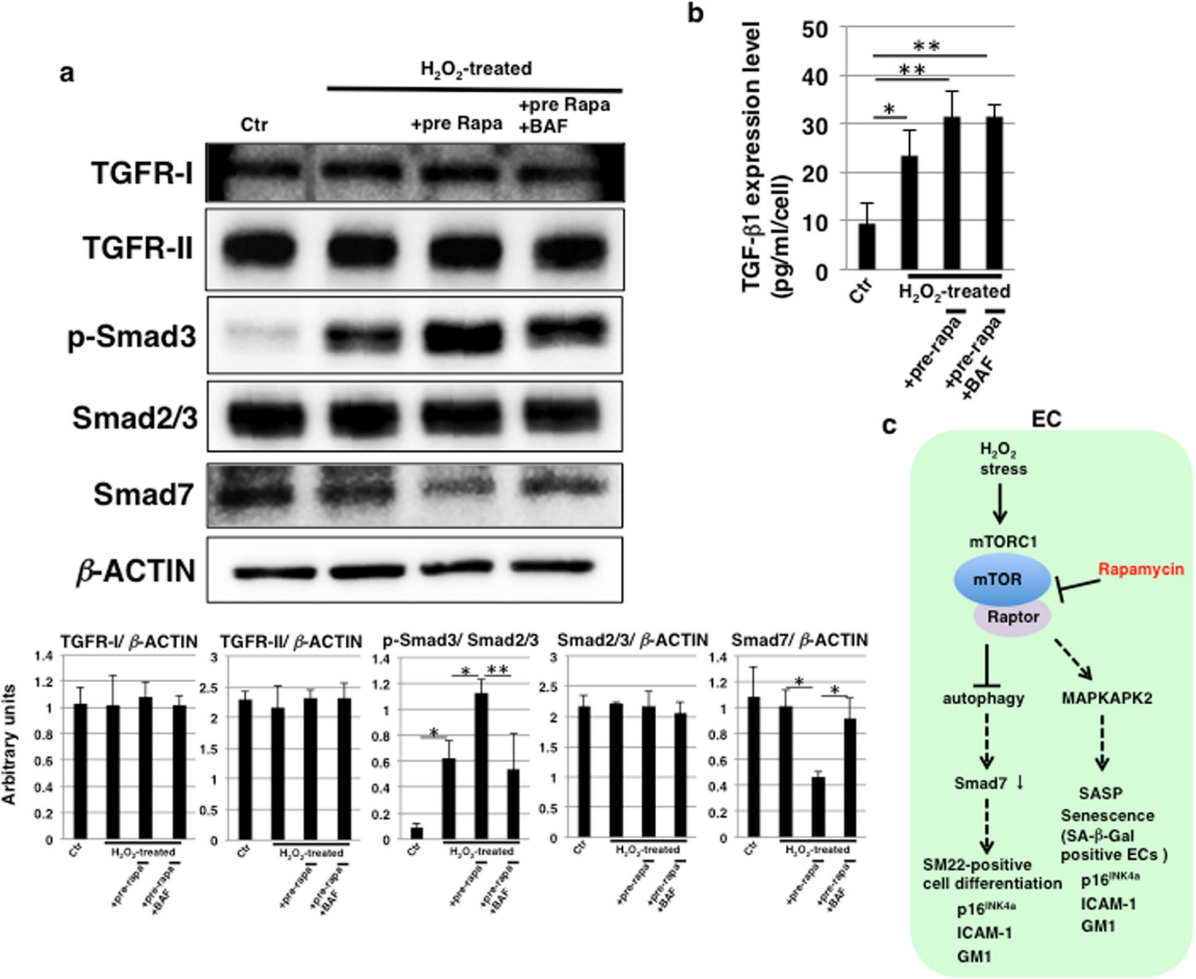
Rapamycin induces TGF-β pathway activation through autophagy activation. **a** Immunoblottings for TGFβR-I, TGFβR-II, p-Smad3, Smad2/3, Smad7 and β-ACTIN were performed with cell lysates on day 1 in Fig. 5a, and representative data are shown. The histogram (lower) shows mean densitometric readings for the proteins normalized to Smad2/3 or β-ACTIN from three independent experiments. *p < 0.05; **p < 0.01. **b** Cell culture supernatant on day 1 in Fig. 5a was subjected to ELISA detection for TGF-β1 levels. Data are expressed as mean ± SD from three independent experiments. *p < 0.05; **p < 0.01. **c** Diagram summarizing the effects of rapamycin on endothelial cells undergoing stress-induced premature senescence (SIPS). Control (Ctr): untreated cells, bafilomycin A1 (BAF). SASP, senescence-associated secretory phenotype

TGF-β signaling has been implicated in EndMT [49]. Accordingly, we hypothesized that the activation of autophagy by rapamycin treatment contributes to TGF-β pathway activation. We found that the expression levels of R-Smads (Smad2/3) were not affected but that Smad3 phosphorylation was enhanced by rapamycin treatment and attenuated by autophagy inhibition (Fig. 6a). These results indicate that the activation of autophagy by rapamycin treatment contributes to TGF-β pathway activation. We next examined how the activation of autophagy influences the TGF-β pathway. The expression levels of TGF-β receptors, TGFβR-I and TGFβR-II, were not affected by H_2_O_2_ stress and rapamycin treatment (Fig. 6a). Then, we investigated whether expression of the I-Smad (Smad7), which acts as a suppressor of the TGF-β pathway, was reduced. As shown in Fig. 6a, the expression levels of Smad7 were downregulated by rapamycin treatment and restored by the inhibition of autophagy. We further examined extracellular levels of TGF-β1. ELISA analysis showed that the levels of released extracellular TGF-β1 were increased 24 h after H_2_O_2_ stress and that the increased levels were not significantly affected by rapamycin treatment and autophagy inhibition (Fig. 6b). Taken together, these results suggest that the induction of autophagy is linked to TGF-β pathway activation by regulating the expression of Smad7 in HCAECs undergoing SIPS.

## Discussion

In this study, rapamycin was found to inhibit SA-β-Gal activity but not the expression of *p16*^*INK4a*^ in HCAECs subject to SIPS (Fig. 1). Furthermore, we showed that the expression of *p16*^*INK4a*^ was correlated with EndMT via the activation of autophagy (Fig. 5 and Additional file 1: Figure S2b). Laberge et al. [38] and Herranz et al. [39] showed that the inhibition of mTOR by rapamycin selectively inhibits the SASP and SA-β-Gal staining of senescent cells without affecting cell cycle arrest. These findings imply that during cell senescence, cell cycle arrest, and the SASP are not coupled and, therefore, can be regulated independently. Thus, our findings suggest that EndMT in HCAECs undergoing SIPS through the activation of autophagy via mTORC1 inhibition is involved in the expression of *p16*^*INK4a*^.

The increase in ICAM-1 and GM1 mediated by rapamycin treatment was suggested to correlate with EndMT in HCAECs (Fig. 5 and Additional file 1: Figure S3). It has been demonstrated that ICAM-1 is increased with EndMT in HUVECs [50]. In that report, at the individual level, EndMT was shown to contribute to the promotion of atherosclerosis and the increase in ICAM-1 was found to be associated with EndMT in atherosclerotic plaques; it is thus thought that a further influx of inflammatory cells via ICAM-1 leads to the progression of atherosclerosis. GM1 is a major component of lipid rafts and is important for the activities of raft-associated proteins [51–53], and the function of ICAM-1 is known to be raft-dependent [54, 55]. The involvement of GM1 in the influx of inflammatory cells via ICAM-1 is unclear; however, it is possible that GM1 is synergistically related to the function of ICAM-1. Therefore, our results using HCAECs predict that the pathology in the coronary arteries would be worsened by rapamycin. However, GM1 was increased and inversely correlated with EndMT in HCAECs treated with bafilomycin A1 (Fig. 5 and Additional file 1: Figure S3), which is a late-autophagy inhibitor that suppresses the fusion of autophagosomes and lysosomes. Regarding GM1, GM1 gangliosidosis is known as a lysosomal disease, and GM1 accumulates at the cell surface and in the lysosomes of cells due to deficiencies in lysosomal degradation enzymes [56, 57]. Here, the increase in GM1 by bafilomycin A1 treatment was suggested to be due to the inhibition of lysosome function, and this situation might be similar to GM1 gangliosidosis. Therefore, it is possible that accumulated GM1 could also appear on the cell surface. The relationship between GM1 synthesis and autophagy requires further study.

In HUVECs, it has been reported that rapamycin induces EndMT through TGF-β pathway activation and that IL-1β promotes EndMT [58]. In that report, it was suggested that rapamycin can activate TGF-β signaling through FK506 binding protein 12 (FKBP12) displacement from the TGF receptor. We also observed altered morphological features and increased expression of the EndMT marker, *SM22* in HCAECs treated only with rapamycin and cultured for 72 h (Additional file 1: Figure S4). But, the increased expression was lower than in combination with H_2_O_2_ stress (Additional file 1: Figure S4). From this result, we suggest that an increase in released extracellular TGF-β1 after H_2_O_2_ stress (Fig. 6b) may be effective on EndMT promotion. Furthermore, we showed here that rapamycin promotes EndMT through the activation of autophagy in HCAECs subjected to SIPS, which is accompanied by the repression of SASP factors including IL-1β. Thus, we speculate that the activation of autophagy, as well as FKBP12 displacement from the TGF receptor, via rapamycin treatment, contributes to EndMT promotion. In glioma cells, the activation of autophagy by rapamycin treatment increases the expression levels of Smad2/3 and decreases the expression levels of Smad7, leading to TGF-β pathway activation [59]. In our study, no effects were observed with respect to the expression levels of Smad2/3 but Smad7 was reduced in rapamycin-treated HCAECs undergoing SIPS. We consider that protein degradation systems might be different in diverse cell types and could be cellular context-dependent. Therefore, further study is required to clarify the mechanisms underlying EndMT differentiation through protein degradation systems including autophagy.

The beneficial effects of rapamycin on the vascular system have been demonstrated at the mouse individual level. In mice fed a high-fat diet, which is accompanied by increased vascular senescence and vascular dysfunction, rapamycin prevents vascular senescence and reduces the severity of limb necrosis and ischemic stroke [60]. Lesniewski et al. [61] showed that dietary rapamycin treatment improves age-related vascular dysfunction including endothelium-dependent dilation and increases the aortic pulse-wave velocity in aged mice. In these reports, the relationship with autophagy is unknown. To date, many preclinical studies on the relationship between autophagy and cardiovascular diseases have been performed [62, 63]. From those studies, it has been suggested that autophagy plays a dual role in cardiovascular disease progression, acting in either beneficial or maladaptive ways, depending on the context. In this study, we showed that the activation of autophagy contributes to the promotion of EndMT. It is known that EndMT is involved in cardiovascular diseases including atherosclerosis, pulmonary hypertension, valvular disease, and fibroelastosis [41, 42, 50]. Therefore, in cardiovascular tissue, whereas the positive effect of rapamycin has been shown, there is also a possibility that the activation of autophagy by rapamycin might be detrimental with respect to EndMT-related pathologies involving atherosclerosis.

## Conclusion

In HCAECs undergoing SIPS, H_2_O_2_ stress induces assembly of mTORC1 via mTOR, and signal transduction leads to MAPKAPK2 translation resulting in induction of the SASP and SA-β-Gal-positive senescence accompanied by increased ICAM-1 and GM1 expression (Fig. 6c). In contrast, rapamycin treatment reduces the assembly of mTORC1 via mTOR inhibition and suppresses the SASP and SA-β-Gal-positive senescence via the attenuation of MAPKAPK2 expression. Moreover, the increased level of expression of *p16*^*INK4a*^, which was found to be related to EndMT by autophagy, in SIPS was maintained even after rapamycin treatment. In addition, the reduced assembly of mTORC1 via the inhibition of mTOR induces the activation of autophagy and TGF-β pathways by reducing Smad7 through autophagy activation, leading to EndMT, which is accompanied by increased expression of ICAM-1 and GM1 (Fig. 6c). In humans, the clinical applications of rapamycin including the elution with rapamycin stent have shown many adverse side effects such as stomatitis, myocardial infarction, heart failure, and hypotension. Although reports of side effects to blood vessels are not well known, adequate caution is required for the clinical application of rapamycin. From the results of our study, we propose that rapamycin should be used in combination with an early-stage autophagy inhibitor, which is not involved in lysosome inhibition, and that other SASP inhibitors need to be developed to prevent aging-related vascular diseases.

## Supplementary information

**Additional file 1: Figure S1.** In rapamycin-pretreated human coronary artery endothelial cells (HCAECs), the attenuation of mTOR signaling is followed by reductions in *IL-1α* and MAPKAPK2 expression. (**a**) Immunoblotting for the levels of mTOR, RICTOR, RAPTOR, and MAPKAPK2 was performed on cell lysates and mTOR immunoprecipitates from HCAECs at 24 h, as in Fig. 1a. (**b**) Real-time PCR analysis of *IL-1α* using cDNA derived from HCAECs at 24 h, as in Fig. 1a. The results are shown after normalization to values obtained from control HCAECs (value = 1). Results are presented as means ± SD from three independent experiments. **p < 0.01. Control (Ctr): untreated cells. **Figure S2.** Pretreatment with bafilomycin A1 has no effect on the repression of SA-β-Gal activity and senescence-associated secretory phenotype (SASP) repression, but attenuates the increased expression of *p16*^*INK4a*^ in rapamycin-treated human coronary artery endothelial cells (HCAECs) subjected to stress-induced premature senescence (SIPS). (**a**) HCAECs at 72 h in Fig. 5a were stained for SA-β-Gal activity. Representative images of staining for SA-β-Gal and DAPI are shown. (**b**) Real-time PCR analysis of *p16*^*INK4a*^ using cDNA derived from HCAECs at 72 h, as in Fig. 5a. The results are shown after normalization to values obtained from control HCAECs (value = 1). Results are presented as means ± SD from three independent experiments. **p < 0.01. (**c**) Real-time PCR analysis of SASP markers using cDNA derived from HCAECs at 72 h, as in Fig. 5a. The results are shown after normalization to values obtained from control HCAECs (value = 1). Results are presented as means ± SD from three independent experiments. ^a^*p* < 0.05 vs. the Ctr and ^b^*p* < 0.05 vs. the H_2_O_2_-treated HCAECs. Control (Ctr): untreated cells, bafilomycin A1 (BAF). **Figure S3.** Autophagy inhibition suppresses the increase in ICAM-1 induced by rapamycin treatment and enhances the increase in GM1. (**a**) FACS analysis of cell surface ICAM-1 and GM1 in human coronary artery endothelial cells (HCAECs) at 72 h, as in Fig. 5a. Mean fluorescence intensities (MFIs) relative to those in control HCAECs based on three independent experiments are shown. *p < 0.05; **p < 0.01. (**b**) Immunocytochemical staining performed on HCAECs at 72 h, as in Fig. 5a. Representative images are shown (ICAM-1, green; SM22, red; GM1, blue; DAPI, grey). Control (Ctr): untreated cells, bafilomycin A1 (BAF). **Figure S4.** Only rapamycin treatment modulates endothelial cell morphological features. (a) Representative photos of human coronary artery endothelial cells (HCAECs) cultured for 72 h with or without rapamycin treatment. (b) Real-time PCR analysis of endothelial–mesenchymal transition (EndMT) markers using cDNA derived from HCAECs at 72 h. The results are shown after normalization to values obtained from control HCAECs (value = 1). Results are presented as means ± SD from three independent experiments. **p < 0.01. Control (Ctr): untreated cells.

## Data Availability

The datasets used and/or analysed during the current study available from the corresponding author on reasonable request.

## Abbreviations

EC: Endothelial cell
SASP: Senescence-associated secretory phenotype
TOR: Target of rapamycin
SA-β-Gal: Senescence-associated β-galactosidase
HUVEC: Human umbilical vein EC
SIPS: Stress-induced premature senescence
HCAEC: Human coronary artery endothelial cell
mTOR: Mammalian TOR
mTORC: mTOR complex
EndMT: Endothelial–mesenchymal transition
FACS: Fluorescence-activated cell sorting
ICAM-1: Intercellular adhesion molecule-1
SMC: Smooth muscle cell
MFIs: Mean fluorescence intensities
