# Supplementary material for: Rapamycin promotes endothelial–mesenchymal transition during stress-induced premature senescence through the activation of autophagy
Source: Cell Commun Signal. 2020 Mar 12;18:43. doi: 10.1186/s12964-020-00533-w (PMC7069020; doi:10.1186/s12964-020-00533-w)
Supplement: Supplementary file 2 — Additional file 1: Figure S1. In rapamycin-pretreated human coronary artery endothelial cells (HCAECs), the attenuation of mTOR signaling is followed by reductions in IL-1α and MAPKAPK2 expression. (a) Immunoblotting for the levels of mTOR, RICTOR, RAPTOR, and MAPKAPK2 was performed on cell lysates and mTOR immunoprecipitates from HCAECs at 24 h, as in Fig. 1a. (b) Real-time PCR analysis of IL-1α using cDNA derived from HCAECs at 24 h, as in Fig. 1a. The results are shown after normalization to values obtained from control HCAECs (value = 1). Results are presented as means ± SD from three independent experiments. **p < 0.01. Control (Ctr): untreated cells. Figure S2. Pretreatment with bafilomycin A1 has no effect on the repression of SA-β-Gal activity and senescence-associated secretory phenotype (SASP) repression, but attenuates the increased expression of p16INK4a in rapamycin-treated human coronary artery endothelial cells (HCAECs) subjected to stress-induced premature senescence (SIPS). (a) HCAECs at 72 h in Fig. 5a were stained for SA-β-Gal activity. Representative images of staining for SA-β-Gal and DAPI are shown. (b) Real-time PCR analysis of p16INK4a using cDNA derived from HCAECs at 72 h, as in Fig. 5a. The results are shown after normalization to values obtained from control HCAECs (value = 1). Results are presented as means ± SD from three independent experiments. **p < 0.01. (c) Real-time PCR analysis of SASP markers using cDNA derived from HCAECs at 72 h, as in Fig. 5a. The results are shown after normalization to values obtained from control HCAECs (value = 1). Results are presented as means ± SD from three independent experiments. ap < 0.05 vs. the Ctr and bp < 0.05 vs. the H2O2-treated HCAECs. Control (Ctr): untreated cells, bafilomycin A1 (BAF). Figure S3. Autophagy inhibition suppresses the increase in ICAM-1 induced by rapamycin treatment and enhances the increase in GM1. (a) FACS analysis of cell surface ICAM-1 and GM1 in human coronary artery e [file 12964_2020_533_MOESM1_ESM.docx]

**Figure S1.** In rapamycin-pretreated human coronary artery endothelial cells (HCAECs), the attenuation of mTOR signaling is followed by reductions in *IL-1α* and MAPKAPK2 expression. (**a**) Immunoblotting for the levels of mTOR, RICTOR, RAPTOR, and MAPKAPK2 was performed on cell lysates and mTOR immunoprecipitates from HCAECs at 24 h, as in Fig. 1a. (**b**) Real-time PCR analysis of *IL-1α* using cDNA derived from HCAECs at 24 h, as in Fig. 1a. The results are shown after normalization to values obtained from control HCAECs (value = 1). Results are presented as means ± SD from three independent experiments. ***p* < 0.01. Control (Ctr): untreated cells.

**Figure S2.** Pretreatment with bafilomycin A1 has no effect on the repression of SA-β-Gal activity and senescence-associated secretory phenotype (SASP) repression, but attenuates the increased expression of *p16^INK4a^* in rapamycin-treated human coronary artery endothelial cells (HCAECs) subjected to stress-induced premature senescence (SIPS). (**a**) HCAECs at 72 h in Fig. 5a were stained for SA-β-Gal activity. Representative images of staining for SA-β-Gal and DAPI are shown. (**b**) Real-time PCR analysis of *p16^INK4a^* using cDNA derived from HCAECs at 72 h, as in Fig. 5a. The results are shown after normalization to values obtained from control HCAECs (value = 1). Results are presented as means ± SD from three independent experiments. ***p* < 0.01. (**c**) Real-time PCR analysis of SASP markers using cDNA derived from HCAECs at 72 h, as in Fig. 5a. The results are shown after normalization against values obtained for control HCAECs (value = 1). Results are presented as means ± SD from three independent experiments. ^a^*p* < 0.05 vs. the Ctr and ^b^*p* < 0.05 vs. the H_2_O_2_-treated HCAECs. Control (Ctr): untreated cells, bafilomycin A1 (BAF).

**Figure S3.** Autophagy inhibition suppresses the increase in ICAM-1 induced by rapamycin treatment but enhances the increase in GM1. (**a**) FACS analysis of cell surface ICAM-1 and GM1 in human coronary artery endothelial cells (HCAECs) at 72 h, as in Fig. 5a. Mean fluorescence intensities (MFIs) relative to those in control HCAECs based on three independent experiments are shown. **p* < 0.05; ***p* < 0.01. (**b**) Immunocytochemical staining performed on HCAECs at 72 h, as in Fig. 5a. Representative images are shown (ICAM-1, green; SM22, red; GM1, blue; DAPI, grey). Control (Ctr): untreated cells, bafilomycin A1 (BAF).

**Figure S4.** Only rapamycin treatment modulates endothelial cell morphological features. (a) Representative photos of human coronary artery endothelial cells (HCAECs) cultured for 72 h with or without rapamycin treatment. (b) The real-time PCR analysis of endothelial–mesenchymal transition (EndMT) markers using cDNA derived from HCAECs at 72 h. The results are shown after normalization to values obtained from control HCAECs (value = 1). Results are presented as means ± SD from three independent experiments. ***p* < 0.01. Control (Ctr): untreated cells.
